# Strategies to assess and optimize stability of endogenous amines during cerebrospinal fluid sampling

**DOI:** 10.1007/s11306-018-1333-0

**Published:** 2018-03-05

**Authors:** Marek J. Noga, Ronald Zielman, Robin M. van Dongen, Sabine Bos, Amy Harms, Gisela M. Terwindt, Arn M. J. M. van den Maagdenberg, Thomas Hankemeier, Michel D. Ferrari

**Affiliations:** 1 Division of Analytical Biosciences, Leiden Academic Centre for Drug Research, Leiden University, Einsteinweg 55, 2333 CC Leiden, The Netherlands; 2 Department of Neurology, Leiden University Medical Centre, Leiden, The Netherlands; 3 Department of Human Genetics, Leiden University Medical Centre, Leiden, The Netherlands

**Keywords:** Cerebrospinal fluid, Stability, Metabolomics, Glutamic acid, Glutamine, Amines

## Abstract

**Introduction:**

Metabolic profiling of cerebrospinal fluid (CSF) is a promising technique for studying brain diseases. Measurements should reflect the in vivo situation, so ex vivo metabolism should be avoided.

**Objective:**

To investigate the effects of temperature (room temperature vs. 4 °C), centrifugation and ethanol, as anti-enzymatic additive during CSF sampling on concentrations of glutamic acid, glutamine and other endogenous amines.

**Methods:**

CSF samples from 21 individuals were processed using five different protocols. Isotopically-labeled alanine, isoleucine, glutamine, glutamic acid and dopamine were added prior to sampling to trace any degradation. Metabolomics analysis of endogenous amines, isotopically-labeled compounds and degradation products was performed with a validated LC–MS method.

**Results:**

Thirty-six endogenous amines were quantified. There were no statistically significant differences between sampling protocols for 31 out of 36 amines. For GABA there was primarily an effect of temperature (higher concentrations at room temperature than at 4 °C) and a small effect of ethanol (lower concentrations if added) due to possible degradation. *O*-phosphoethanolamine concentrations were also lower when ethanol was added. Degradation of isotopically-labeled compounds (e.g. glutamine to glutamic acid) was minor with no differences between protocols.

**Conclusion:**

Most amines can be considered stable during sampling, provided that samples are cooled immediately to 4 °C, centrifuged, and stored at − 80 °C within 2 h. The effect of ethanol addition for more unstable metabolites needs further investigation. This was the first time that labeled compounds were used to monitor ex vivo metabolism during sampling. This is a useful strategy to study the stability of other metabolites of interest.

**Electronic supplementary material:**

The online version of this article (10.1007/s11306-018-1333-0) contains supplementary material, which is available to authorized users.

## Introduction

Novel high-throughput biochemical analysis technologies used in metabolomics and proteomics allow for large-scale profiling of metabolites and proteins in body fluids (Baggerman et al. 2004; Patti et al. 2012; Schutzer et al. 2010). With their advent there is growing interest in using cerebrospinal fluid (CSF) as a relatively easy accessible source for the discovery of biomarkers reflecting biochemical changes in the dysfunctional brain (Zhang et al. 2013). Wide-scale “CSF-omics” studies aim to reveal novel biochemical pathways for brain diseases (Mitchell et al. 2009; Nishino et al. 2001), and to provide diagnostic (Duits et al. 2014; Spies et al. 2013) and prognostic biomarkers (Hansson et al. 2006; Stewart et al. 2014).

In addition to the technological challenge to continuously increase coverage, sensitivity, specificity and throughput of the analytical methods, stability of the metabolites during and after sampling is of major importance (Anesi et al. 1998; Levine et al. 2000), but largely ignored. After all, ex vivo changes of unstable compounds could occur due to enzymatic or chemical reactions (Anesi et al. 1998; Del Campo et al. 2012; Schoonenboom et al. 2005). In clinical practice, CSF is often sampled using protocols designed for routine clinical measurements (i.e. cell count, total protein, glucose) and not specifically for metabolomics or proteomics. Samples are primarily transported at room temperature and time until analysis and subsequent storage of remaining CSF in the freezer can vary significantly. This increases the risk of ex vivo biochemical changes that may occur after withdrawal of the CSF, and potentially reducing the comparability with samples from other studies when non-routine measurements such as metabolomics or proteomics are performed (Vanderstiechele et al. 2012).

Previously published consensus guidelines for CSF sampling have already established some main pre-analytical factors that should be standardized (Del Campo et al. 2012; Teunissen et al. 2009; Vanderstiechele et al. 2012). However, the scientific basis for this guideline was mainly based on stability studies that focused on protein biomarkers (for Alzheimer’s disease and Parkinson’s disease), i.e. these guidelines do not have recommendations for samples used for metabolomics studies. For the CSF metabolome, major factors that can cause ex vivo biochemical changes include the temperature during sampling and processing, additional procedures such as centrifugation to remove cells, and the use of additives to stabilize the metabolic profile (Del Campo et al. 2012). It is especially important to determine these factors for metabolites which function as neurotransmitter or neurotransmitter precursors, since these are of primary interest for most brain disorders. Concentrations of glutamic acid, the major excitatory neurotransmitter of the brain (Danbolt 2001), could be unstable in CSF because of potential ex vivo degradation of its precursor glutamine (Anesi et al. 1998; Ferrarese et al. 1993). However, so far this degradation has not been quantified.

The aim of this study was to investigate the effect of major sample handling factors (low sampling and processing temperature, centrifugation to remove cells, and addition of ethanol to stop enzymatic reactions) on the stability of the metabolic profile of primary and secondary endogenous amines. To this end we compared five different sample handling protocols for CSF. In addition, we quantified the degradation of allegedly unstable metabolites such as glutamine by adding isotopically-labeled versions of these metabolites to sampling tubes before CSF sampling.

## Materials and methods

### CSF sampling procedure

We obtained human CSF samples from 21 individuals (16 migraine patients and 5 healthy controls) as part of a research program on migraine pathophysiology. The study was approved by the Medical Ethical Committee of Leiden University Medical Centre (LUMC). All subjects gave written informed consent prior to collection.

CSF sampling was performed before 12.00 am via lumbar puncture. All subjects were overnight fasted and only allowed water in the 8 h preceding lumbar puncture. Local skin was disinfected with Chlorhexidine (5 g/L)/denatured ethanol 70% (art.no. 909602; Pharmacy LUMC, Leiden, the Netherlands). CSF was sampled between the L3/L4, L4/L5 or L5–S1 interspace with an atraumatic Sprotte® needle (Pajunk GmbH, Geisingen, Germany). For routine CSF diagnostics we collected 3 mL CSF followed by 12 mL for migraine research purposes.

### Five different sampling protocols

**Fig. 1:**
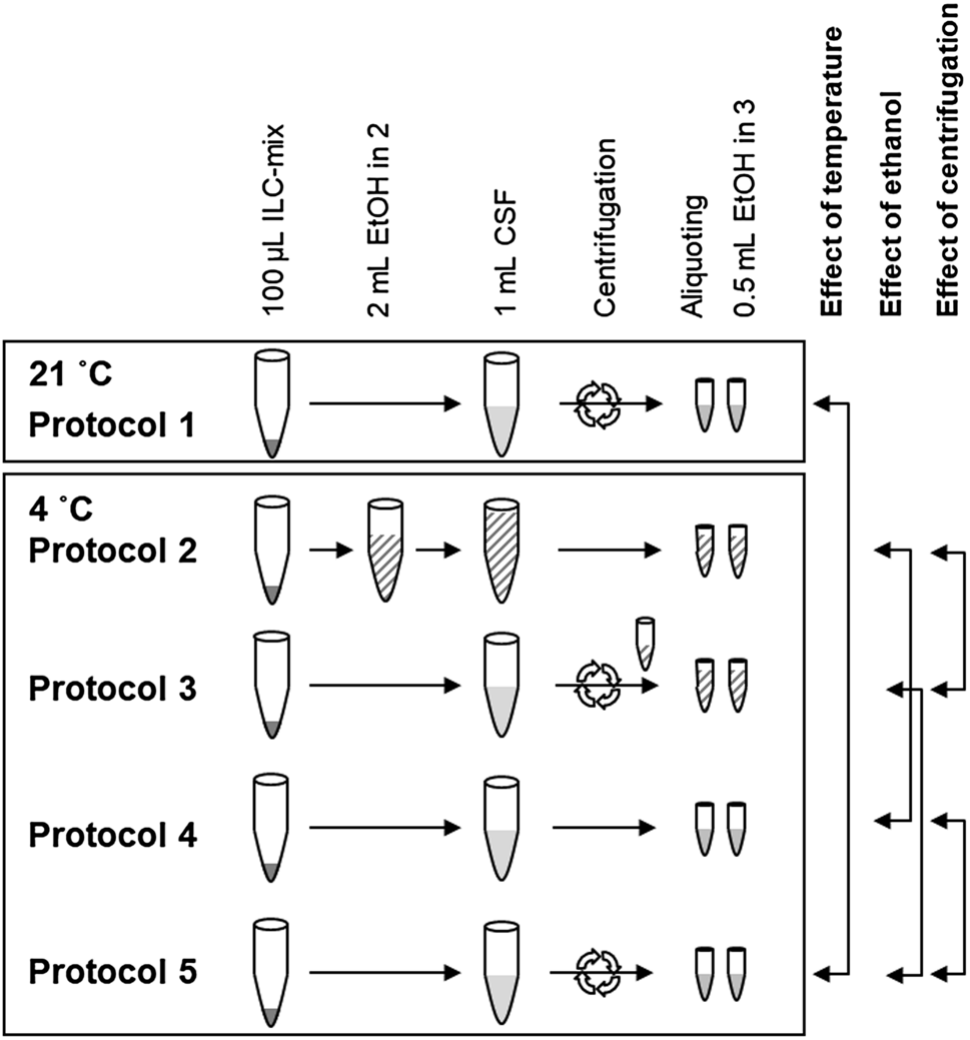
Scheme of different CSF handling protocols. Temperature was different for protocol 1. Ethanol was added in protocols 2 and 3 (striped pattern). Centrifugation was performed in protocols 1, 3 and 5 (arrows). *ILC-mix* mix of isotopically-labeled compounds; *EtOH* ethanol

For this study we additionally collected five times 1 mL CSF in five separate 15-mL polypropylene falcon tubes (art.no. 188271; Greiner Bio-One, Kremsmünster, Austria) that already contained a 100-µL mix of isotopically-labeled compounds (ILC-mix; preparation described in 2.3). Tubes were inverted at least ten times to mix the CSF and ILC-mix. For each of the five tubes there was a unique sample handling protocol to study the effects of temperature, centrifugation and the addition of ethanol. The five protocols are depicted in Fig. 1.

For protocol 1, CSF collection and centrifugation were performed at room temperature; for the additional four protocols sampling tubes were prepared on ice before CSF was added and further sample handling was also done on ice. For protocol 2, the sampling tube also contained 2 mL cold ethanol (ethanol absolute prod. no. 8098; J.T.Baker, Avantor Performance Materials, Center Valley, PA, USA) besides the ILC-mix. For protocol 3, centrifugation was performed and ethanol was later added during aliquoting. For protocol 4, we performed no additional sample handling steps. For protocol 5, the samples were centrifuged.

All centrifugation steps (protocols 1, 3, and 5) were performed for 5 min (2000 rpm, 747 g) directly after sampling. Centrifugation in protocol 1 was performed at 21 °C and for protocols 3 and 5 at 4 °C. After centrifugation, the supernatant was transferred to another 15-mL polypropylene falcon tube, and divided in aliquots. Samples from protocols 1, 4 and 5 were divided in 0.25-mL aliquots [into 0.5-mL cryotubes (Eppendorf, Hamburg, Germany)], samples from protocols 2 and 3 in 0.75-mL aliquots [into 1.5-mL cryotubes (Eppendorf)] because of the additional ethanol. All aliquots were immediately placed on dry ice after processing, within 30–60 min from sampling, and transferred to − 80 °C for storage within 120 min from sampling. All samples remained at − 80 °C until sample preparation, no extra freeze–thaw cycles were allowed.

### Compound labeling strategy

To assess whether potential differences between protocols were due to metabolic degradation, and not due to other factors, we added ILC-mix containing two stable and three potentially unstable compounds to the collection tubes. Stable isotopically-labeled compounds were: 2,3-^13^C_2_-alanine (ILC-mix: 300 µmol/L; Cortecnet, Voisins-Le-Bretonneux, France) and ^13^C^15^N-isoleucine (ILC-mix: 30 µmol/L; Cambridge isotope laboratories, Tewksbury, MA, USA) (Davis et al. 2009). Potentially unstable compounds were: 1,3-^13^C_2_-glutamine (ILC-mix: 5000 µmol/L; Cambridge isotope laboratories, Tewksbury, MA, USA), U^13^C–U^15^N–UD-glutamic acid (ILC-mix: 125 µmol/L; Cambridge isotope laboratories, Tewksbury, MA, USA), and 1,2,3,4,5,6-^13^C_6_-dopamine (ILC-mix: 1.25 µmol/L; CDN Isotopes, Pointe-Claire, Canada). Isotopically-labeled compounds were selected to minimize overlap of their isotopic envelopes. Concentrations of isotopically-labeled compounds were chosen such that they approximate physiological concentrations after addition of CSF. The ILC-mix was prepared in one batch, then divided into aliquots, and stored at − 80 °C until the day of sampling. Maximal 1 h before sampling the ILC-mix was defrosted at room temperature. For each subject, we prepared the five 15-mL polypropylene collection tubes for sampling by adding 100 µL of the ILC-mix.

### Measurements of endogenous amines

For the targeted analysis of primary and secondary amines we employed a LC–MS method using AccQ-Tag derivatization as described before (Noga et al. 2012). Measurements were performed with Xevo TQ mass spectrometer (Waters, Etten-Leur, The Netherlands) operating with the width of the isolation/fragmentation window of 1.2 Da. Cell-free U–^13^C–U^15^N-labeled amino acid mix (Cambridge isotope laboratories), D_4_-β-alanine, D6-ornithine, D_4_-histamine, D_6_-2-aminobutyric acid, D_3_-3-methyl-histidine, D_3_-3-methoxytyrosine and D_4_-3-methoxytyramine (CDN Isotopes) were used as internal standards. Acquired data were evaluated using MassHunter software (Agilent, Santa Clara, CA, USA) by integration of assigned SRM peaks and normalization using proper internal standards (ISTDs). For analysis of amino acids, their U–^13^C–U^15^N-labeled analogues were used as ISTDs. For other amines—the closest-eluting ISTD was used. Relative concentrations of endogenous compounds were reported as response ratios to their respective ISTDs. Data quality was monitored and compensated for shifts in sensitivity of the mass spectrometer over time using additionally measured quality control (QC) samples as described before (van der Kloet et al. 2009). Measurement variation was evaluated by calculating the relative standard deviation (RSD) per amine from replicate samples and QC samples.

### Measurements of isotopically-labeled stability markers

Isotopically-labeled stability markers and degradation products were measured with a modified version of the method used for endogenous compounds. Sample preparation and LC settings remained unchanged and the MRM transitions of MS were set for specific masses of isotopically-labeled compounds, including spiked compounds and potential degradation product of ^13^C_2_-glutamine namely ^13^C_2_-glutaminc acid. For normalization, the same ISTDs were used as for the endogenous equivalents. Similarly, the referred QC approach was applied. Calibration samples were prepared with pure standards of isotopically-labeled compounds, spiked at 7 consecutive twofold dilutions into aliquots of CSF pooled from all the study samples. Covered concentration ranges were: 3.75–240 µM for ^13^C_2_-alanine, 0.875–56 µM for ^13^C^15^N-isoleucine, 65.5–4000 µM ^13^C_2_-glutamine, 1.56–100 µM for U–^13^C^15^ND-glutamic acid and 0.0156–1 µM for ^13^C_6_-dopamine HCl. Samples of CSF pool without any standard were used as zero point for the calibration. Absolute concentrations in µmol/L were calculated using linear calibration lines as previously described and implemented in R package ‘chemCal’ (Massart et al. 1997).

### Correction for volume differences

In protocols 2 and 3 the sampling procedure involved mixing CSF and ILC-mix with ethanol. Potential in-accuracies in mixing of different volumes can lead to an increased variability of metabolites concentrations or a systematic bias in further analysis. Especially in protocol 2 the CSF volume is less controlled because CSF drips directly into a relatively large sampling tube already containing ethanol. In CSF samples from protocol 2 concentrations of almost every endogenous and isotopically-labeled metabolite were approximately 20% higher in comparison to samples from the other protocols (Fig. S-1). The fact that both endogenous (from CSF) and isotopically-labeled metabolites (from ILC-mix) were increased indicated that there was less ethanol in samples from protocol 2 than anticipated. This systematic error could result from evaporation of ethanol before, during and after sampling, or might be caused by inconsistencies in pipetting of cooled volatile ethanol.

In order to test for differences in endogenous metabolite levels between all five protocols, without the influence of the systematic error described above, we standardized concentrations of all endogenous metabolites to the concentrations of endogenous l-alanine and l-isoleucine (Fig. S-1). l-alanine and l-isoleucine are compounds with hardly any degradation ex-vivo (Davis et al. 2009). Concentrations of all isotopically-labeled compounds were standardized to the concentrations of ^13^C_2_-alanine and ^13^C^15^N-isoleucine in a special reference sample (ILC-mix with 1 mL of water instead of CSF, sampled and processed on the same day as clinical samples). See Supplemental Methods for full descriptions and examples of the applied corrections.

### Statistical analysis

Relative concentrations of endogenous compounds and absolute concentrations of stability markers were log-transformed prior to statistical analysis (Bland and Altman 1996; Sumner et al. 2007). To test for differences between the five protocols, we applied one-way repeated measures ANOVA. Reported p-values from repeated measures ANOVA were not corrected for multiple testing. P-values below 0.05 were considered significant. When significant, post-hoc pairwise *t*-test comparisons were applied with Bonferroni’s correction. The same ANOVA strategy was used to compare degradation percentages of ^13^C_2_-glutamine to ^13^C_2_-glutamic acid (volume correction and log-transformation were not necessary for these data). Repeated measures ANOVA was performed with R software (package “ez” version 4.2-2).

## Results

### Sample set

We obtained CSF from 12 females and 9 males (mean age of 38.2 ± 12.2 years old). Routine CSF diagnostics were performed and were all within reference limits. Median erythrocyte count was 2.5 cells/3 µL (range 0–200); mean leucocyte count 3.50 ± 2.50 cells/3 µL; mean total protein 0.33 ± 0.11 g/L; and mean glucose levels 3.24 ± 0.19 mmol/L. With LC–MS we identified and quantified 36 amino acids and 7 isotopically-labeled compounds.

### Endogenous metabolites

**Fig. 2:**
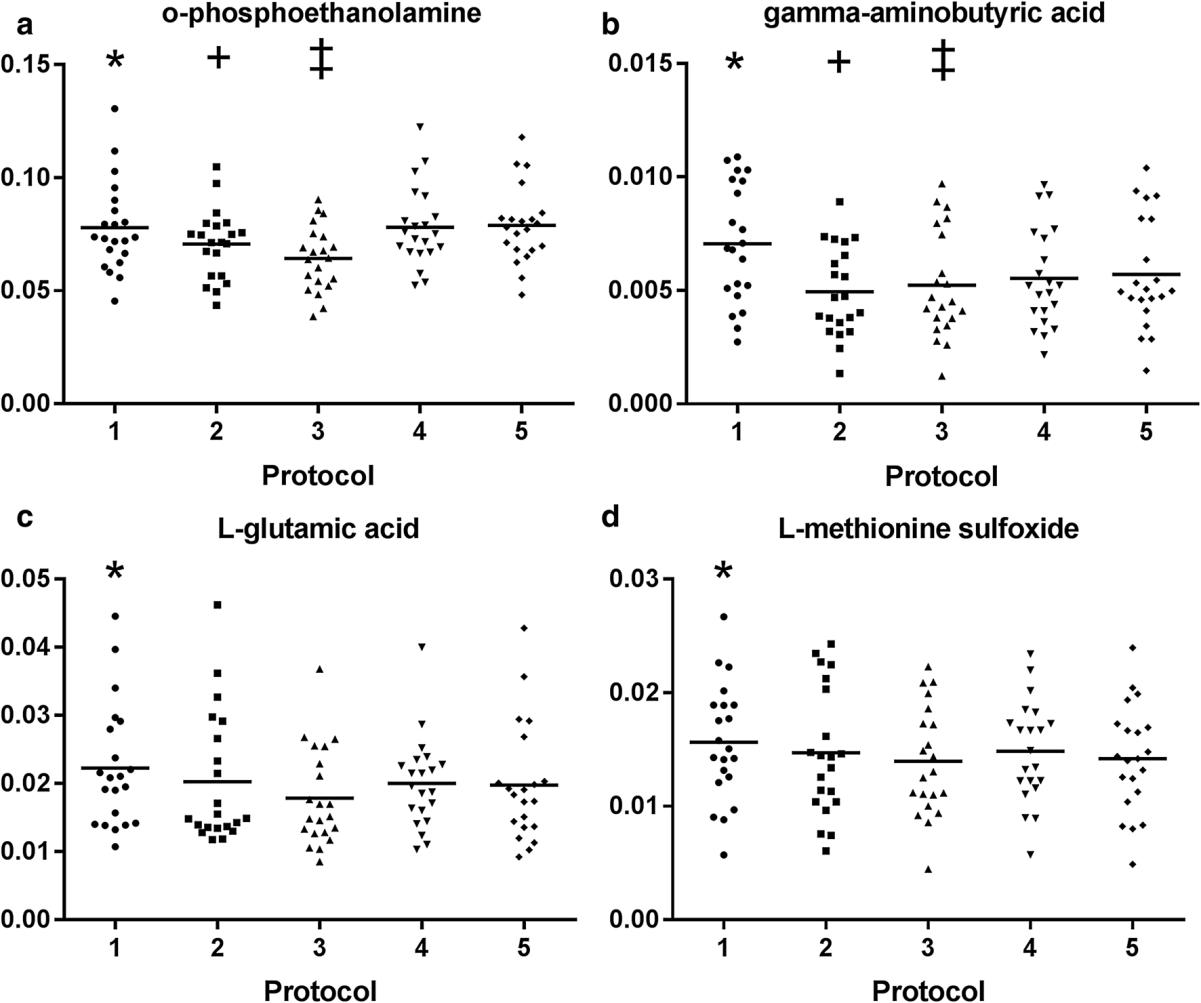
Scatter plots of metabolites with significant protocol differences. Relative concentrations reported as response ratios to their respective internal standards. **a** *O*-phosphoethanolamine: significant differences between protocol 1 (*) and protocol 3 (p-value = 0.007); between protocol 2 (*) and protocols 4 (p-value = 0.041) and 5 (p-value = 0.006); and between protocol 3 (‡) and protocols 4 (p-value = 0.001) and 5 (p-value < 0.001). **b** Gamma-aminobutyric acid: significant differences between protocol 1 (*) and protocols 2–5 (p-value < 0.001 for all comparisons); between protocol 2 (+) and protocols 4 (p-value = 0.027) and 5 (p-value = 0.014); and between protocol 3 (‡) and protocol 5 (p-value < 0.001). **c** l-glutamic acid: significant difference between protocol 1 (*) and protocol 3 (p-value = 0.004). **d** l-methionine sulfoxide: significant difference between protocol 1 (*) and protocol 3 (p-value = 0.016)

There were no significant concentration differences between the sampling protocols for 31 out of 36 (86.1%) amines. Five amines showed significant differences: *O*-phosphoethanolamine (PE), gamma-aminobutyric acid (GABA), l-glutamic acid, l-methionine sulfoxide and l-aspartic acid (Table 1). PE concentrations were significantly lower in the protocols in which ethanol was used as additive (protocols 2 and 3) compared to protocols without ethanol (protocols 4 and 5; Fig. 2a). GABA concentrations were also lower in samples from protocols 2 and 3 (with ethanol) compared to samples from protocols 1 and 5 (without ethanol; Fig. 2b). However, GABA concentrations were primarily higher in samples processed at room temperature (protocol 1) than in samples processed on ice (protocols 2–5; Fig. 2b). Concentrations of PE, l-glutamic acid and l-methionine sulfoxide concentrations were also higher in protocol 1 (room temperature) but only compared to protocol 3 (on ice plus centrifugation; Fig. 2a, c, d). l-aspartic acid showed no significant differences in the post-hoc analysis (data not shown in Fig. 2). To exclude potential confounding effects of the volume correction, we performed an additional analysis in which we excluded protocol 2 and did not perform volume correction. This analysis showed the same effects for GABA, PE and l-glutamic acid (Table S-1).

**Table 1:** Relative concentrations of endogenous amines per protocol after volume correction

Metabolites	Relative concentration					p-value
	Protocol 1	Protocol 2	Protocol 3	Protocol 4	Protocol 5	
*O*-phosphoethanolamine	0.078 ± 0.02	0.071 ± 0.015	0.064 ± 0.014	0.078 ± 0.018	0.079 ± 0.017	< **0.001**
Gamma-aminobutyric acid	0.0071 ± 0.0026	0.0049 ± 0.002	0.0052 ± 0.0024	0.0055 ± 0.0022	0.0057 ± 0.0024	< **0.001**
l-glutamic acid	0.022 ± 0.009	0.02 ± 0.01	0.018 ± 0.007	0.020 ± 0.007	0.020 ± 0.009	**0.017**
l-methionine sulfoxide	0.016 ± 0.005	0.015 ± 0.006	0.014 ± 0.005	0.015 ± 0.004	0.014 ± 0.005	**0.033**
l-aspartic acid	0.055 ± 0.051	0.052 ± 0.068	0.051 ± 0.048	0.054 ± 0.043	0.050 ± 0.051	**0.047**
l-methionine	0.537 ± 0.123	0.544 ± 0.126	0.554 ± 0.132	0.534 ± 0.121	0.544 ± 0.121	0.124
SDMA	0.0039 ± 0.0009	0.0045 ± 0.0013	0.0044 ± 0.0016	0.0040 ± 0.0011	0.0041 ± 0.0012	0.168
l-isoleucine	0.013 ± 0.004	0.013 ± 0.003	0.013 ± 0.003	0.014 ± 0.004	0.013 ± 0.004	0.172
ADMA	0.0005 ± 0.0002	0.0006 ± 0.0003	0.0007 ± 0.0003	0.0006 ± 0.0003	0.0006 ± 0.0003	0.177
l-alanine	0.068 ± 0.016	0.069 ± 0.018	0.070 ± 0.018	0.068 ± 0.016	0.068 ± 0.017	0.208
l-threonine	1.359 ± 0.359	1.356 ± 0.368	1.383 ± 0.385	1.366 ± 0.363	1.396 ± 0.387	0.248
l-asparagine	0.322 ± 0.073	0.332 ± 0.078	0.327 ± 0.081	0.323 ± 0.074	0.326 ± 0.075	0.286
l-serine	2.574 ± 0.665	2.590 ± 0.776	2.625 ± 0.679	2.620 ± 0.636	2.660 ± 0.679	0.362
l-valine	0.037 ± 0.011	0.037 ± 0.011	0.037 ± 0.011	0.036 ± 0.011	0.037 ± 0.011	0.406
l-arginine	1.896 ± 0.458	1.931 ± 0.471	2.005 ± 0.56	1.898 ± 0.472	1.969 ± 0.492	0.432
l-lysine	3.221 ± 0.787	3.283 ± 0.806	3.305 ± 0.913	3.248 ± 0.746	3.306 ± 0.8	0.436
Ethanolamine	3.595 ± 0.562	3.726 ± 0.787	3.784 ± 0.873	3.739 ± 0.69	3.818 ± 0.642	0.447
l-proline	0.025 ± 0.018	0.025 ± 0.018	0.025 ± 0.016	0.025 ± 0.016	0.024 ± 0.016	0.455
Taurine	0.302 ± 0.085	0.320 ± 0.108	0.323 ± 0.132	0.307 ± 0.092	0.315 ± 0.096	0.465
l-kynurenine	0.0023 ± 0.0012	0.0025 ± 0.0016	0.0023 ± 0.0011	0.0024 ± 0.0011	0.0025 ± 0.0009	0.473
N6N6N6-trimethyl-l-lysine	0.0056 ± 0.0008	0.0056 ± 0.0013	0.0059 ± 0.0014	0.0058 ± 0.0011	0.006 ± 0.001	0.474
Putrescine	0.0082 ± 0.0025	0.0085 ± 0.0031	0.0087 ± 0.0035	0.0078 ± 0.0028	0.0084 ± 0.0029	0.526
l-leucine	0.018 ± 0.004	0.018 ± 0.004	0.018 ± 0.004	0.017 ± 0.004	0.018 ± 0.004	0.552
l-2-aminoadipic acid	0.0011 ± 0.0004	0.0011 ± 0.0004	0.0011 ± 0.0004	0.0011 ± 0.0004	0.0011 ± 0.0004	0.563
l-tryptophan	0.310 ± 0.064	0.323 ± 0.085	0.326 ± 0.082	0.312 ± 0.068	0.318 ± 0.065	0.588
Glycylglycine	0.021 ± 0.008	0.021 ± 0.005	0.020 ± 0.009	0.023 ± 0.015	0.022 ± 0.008	0.595
l-histidine	0.090 ± 0.016	0.094 ± 0.02	0.094 ± 0.025	0.092 ± 0.018	0.094 ± 0.018	0.651
l-glutamine	11.877 ± 2.061	12.231 ± 2.401	12.471 ± 2.977	12.217 ± 2.571	12.320 ± 2.696	0.665
l-alpha aminobutyric acid	0.072 ± 0.021	0.073 ± 0.023	0.074 ± 0.025	0.072 ± 0.021	0.074 ± 0.023	0.685
l-tyrosine	0.725 ± 0.154	0.751 ± 0.189	0.756 ± 0.219	0.73 ± 0.154	0.747 ± 0.169	0.790
Sarcosine	0.0010 ± 0.0003	0.0009 ± 0.0004	0.0010 ± 0.0005	0.0011 ± 0.0006	0.0011 ± 0.0006	0.852
Ornithine	0.200 ± 0.071	0.198 ± 0.087	0.199 ± 0.069	0.198 ± 0.066	0.199 ± 0.067	0.885
l-phenylalanine	0.023 ± 0.004	0.023 ± 0.005	0.023 ± 0.006	0.022 ± 0.004	0.023 ± 0.005	0.912
l-homoserine	0.0061 ± 0.0014	0.0060 ± 0.0011	0.0059 ± 0.0014	0.0060 ± 0.0017	0.0060 ± 0.001	0.937
Citrulline	0.04 ± 0.014	0.04 ± 0.015	0.04 ± 0.014	0.039 ± 0.014	0.04 ± 0.016	0.941
l-4-hydroxyproline	0.023 ± 0.013	0.024 ± 0.014	0.024 ± 0.014	0.023 ± 0.014	0.024 ± 0.015	0.947

Relative concentrations of endogenous compounds reported as response ratios to their respective internal standards. P-values from one-way repeated measures ANOVA. Metabolites sorted based on p-value (small to large). P-values < 0.05 are depicted in bold

*SDMA* symmetric dimethylarginine, *ADMA* asymmetric dimethylarginine

### Labeled stability markers

There were no significant differences between protocols in concentrations of the stable (^13^C_2_-alanine, and ^13^C^15^N-isoleucine) and the potentially unstable isotopically-labeled markers (^13^C_2_-glutamine, U^13^C–U^15^N–UD-glutamic acid, and ^13^C_6_-dopamine) (Table 2). We observed very low concentrations for ^13^C_2_-glutamic acid, the degradation product of ^13^C_2_-glutamine. Degradation ranged from 0.16 to 0.17% of the original ^13^C_2_-glutamine levels with no difference between protocols. We did not detect degradation products of ^13^C_6_-dopamine. An additional analysis in which we excluded protocol 2 also showed no significant differences between protocols (Table S-2).

**Table 2:** Labeled stability markers per protocol after volume correction

Metabolites	Absolute concentration (µmol/L)					p-value
	Protocol 1	Protocol 2	Protocol 3	Protocol 4	Protocol 5	
Stable markers						
2,3-^13^C_2_-alanine	28.89 ± 0.3	28.77 ± 0.39	28.84 ± 0.41	28.88 ± 0.32	28.77 ± 0.22	0.564
^13^C^15^N-isoleucine	6.22 ± 0.07	6.24 ± 0.09	6.23 ± 0.09	6.22 ± 0.07	6.24 ± 0.05	0.567
Unstable markers						
1,3-^13^C_2_-glutamine	453.6 ± 18.17	467.1 ± 53.57	455.99 ± 50.93	454.01 ± 11.79	450.37 ± 16.62	0.613
^13^C_2_-glutamic acid	0.72 ± 0.06	0.75 ± 0.08	0.72 ± 0.08	0.75 ± 0.07	0.75 ± 0.07	0.101
U^13^C-U^15^N-UD-glutamic acid	11.44 ± 0.36	11.64 ± 0.66	11.32 ± 0.89	11.56 ± 0.30	11.57 ± 0.33	0.125
1,2,3,4,5,6-^13^C_6_-dopamine	0.11 ± 0.01	0.11 ± 0.02	0.11 ± 0.02	0.11 ± 0.01	0.10 ± 0.01	0.508

Metabolites	Conversion from glutamine to glutamic acid (%)					p-value
	Protocol 1	Protocol 2	Protocol 3	Protocol 4	Protocol 5	
Degradation^a^						
1,3-^13^C_2_-glutamine to ^13^C_2_-glutamic acid (%)	0.158 ± 0.012	0.162 ± 0.026	0.160 ± 0.027	0.165 ± 0.015	0.166 ± 0.017	0.346

P-values from one-way repeated measures ANOVA

^a^Volume correction was not necessary since both glutamine and glutamic acid were measured in the same volume

## Discussion

When performing metabolomics in CSF we want our measurements to reflect the in vivo situation as close as possible. It is therefore important to avoid ex vivo metabolism. The aim of this study was to investigate the effect of three major factors (low sampling and processing temperature, centrifugation to remove cells, and the use of ethanol as additive to stop enzymatic reactions) on the stability of primary and secondary endogenous amines. To this end we compared five different CSF sample handling protocols. In addition, we aimed to quantify the degradation of allegedly unstable metabolites such as l-glutamine and l-glutamic acid by adding isotopically-labeled versions of these metabolites to sampling tubes before CSF sampling. We showed that the effects of different protocols were minimal, except for a clear effect of temperature (21 vs. 4 °C) on GABA, and effects of ethanol on PE and partly on GABA. In addition, our labeling strategy with isotopically-labeled compounds allowed us to successfully monitor degradation of l-glutamine to l-glutamic acid and showed that the amount of degradation was extremely minimal, with no difference between protocols.

Our observation that most endogenous amines are stable under controlled sampling conditions with minimal delay in sample storage is in line with other large scale stability studies which have measured multiple amino acids in CSF (Ferraro and Hare 1984; Lundqvist et al. 1989; Rosenling et al. 2009, 2011). In addition, the fact that the reported CSF concentrations of most amines are quite similar over different studies also indicates that most endogenous amines in CSF are stable, despite varying methodologies for sample handling and measurement (Lundqvist et al. 1989). However, glutamic acid is an important exception given that reported concentrations in CSF vary widely between studies (Ferrarese et al. 1993; Lundqvist et al. 1989). Additionally, studies investigating the stability of glutamic acid report varying results which suggests that glutamic acid concentrations might be unstable in CSF (Anesi et al. 1998; Ferrarese et al. 1993).

The ex vivo non-enzymatic (chemical) degradation of l-glutamine to l-glutamic acid is negligible. Studies measuring l-glutamine degradation in water showed that there is only a small amount of non-enzymatic degradation at room temperature (< 1% if left at room temperature for more than 15 days) and no observable decrease at − 80 °C (Khan and Elia 1991; Snowden et al. 2002). More importantly, degradation did not result in the formation of glutamic acid; instead it presumably resulted in formation of pyroglutamic acid. Only after heating to 100 °C, small amounts of glutamic acid were detected (Snowden et al. 2002).

The ex vivo enzymatic conversion of l-glutamine and l-glutamic acid is therefore the most important factor leading to the alleged instability of these compounds (Ferraro and Hare 1984). When left at room temperature, glutamic acid levels in untreated CSF steadily increased and double within 24 h (Ferrarese et al. 1993; Ferraro and Hare 1984; Rosenling et al. 2009, 2011). The time-related glutamic acid changes in CSF suggests enzymatic processes that mediate the slow formation of new glutamic acid from glutamine or proteins (Ferrarese et al. 1993). Different additives have been tried to deproteinize CSF, such as by trichloroacetic acid (Anesi et al. 1998), sulfosalicylic acid (Lakke and Teelken 1976), or perchloric acid (Lundqvist et al. 1989), however, this led to conflicting results because acidic conditions also cause other chemical reactions and release of bound amino acids.

The labeling strategy applied in this study allowed the selective monitoring of the conversion of l-glutamine to l-glutamic acid. We observed almost no degradation from ^13^C_2_-glutamine to ^13^C_2_-glutamic acid. Because the concentration of l-glutamine in CSF is approximately thousand times higher than l-glutamic acid, it is theoretically still possible that minimal degradation of l-glutamine is affecting l-glutamic acid concentrations. However, since we did not observe significant protocol differences for isotopically-labeled compounds (^13^C_2_-glutamine, ^13^C_2_-glutamic acid and U^13^C–U^15^N–UD-glutamic acid) the impact will be similar for all protocols.

The rate of chemical and enzymatic activity is highly dependent on the sample temperature, which combined with a delayed storage determines the actual degree of change (Ferrarese et al. 1993; Ferraro and Hare 1984). The effect of delayed storage was minimal in our study because all samples were placed on dry ice within 1 h and stored at − 80 °C within 2 h. We did observe a clear effect of temperature on GABA concentrations, which were higher at room temperature (protocol 1) compared to samples that were processed at 4 °C (other protocols). The increase of GABA at room temperature is in line with previous reports on GABA stability (Ferraro and Hare 1984; Grossman et al. 1980) and is thought to be secondary to enzymatic hydrolysis of GABA-containing peptides (Hare et al. 1981). We also observed significant higher levels of endogenous l-glutamic acid and l-methionine sulfoxide in samples at room temperature (protocol 1) compared to cooled samples containing ethanol (protocol 3). Taken together, the effect of temperature seems limited for most of the metabolites provided that the CSF samples are processed quickly, cooled to − 20 °C within 1 h and stored at − 80 °C within 2 h. In conclusion, it is advisable to quickly cool CSF samples and perform sample processing at 4 °C to reduce enzymatic activity, which seems especially relevant for GABA.

Immune cells, either native to CSF or artificially introduced due to blood contamination during sampling may be present in CSF (Rosenling et al. 2009). Removing cells is also expected to limit effects of enzymatic and metabolic activity in CSF, and in addition reduces the risk of contamination of CSF with cytoplasmic metabolites due to cell lysis during storage at − 80 °C (Rosenling et al. 2009). In our study we did not observe a clear impact of centrifugation. This might be due to the fact that contamination of CSF samples with blood was minimal, as evidenced by the low red blood cell count in our samples. As even minor contamination can have a major impact on the metabolic profile (Rosenling et al. 2009), we advise a centrifugation step immediately after withdrawal of CSF.

Ethanol was used in this study because it has a non-specific denaturation effect on all enzymes and will reduce or stop their activity; this inactivates most of the metabolic reactions and thereby, in theory, should stabilize the CSF metabolome (Alfredsson and Sedvall 1983; Alfredsson et al. 1988). However, the effect of ethanol for the metabolites analyzed in this study was small; the two protocols containing ethanol (protocols 2 and 3) showed slightly lower concentrations of PE (decrease of 19%) and GABA (decrease of 9%) compared to the other protocols (Table 1). Ex vivo PE and GABA are known as degradation products of phospholipids and GABA-containing peptides, respectively (Grossman et al. 1980; Hare et al. 1981). Sampling into pre-cooled ethanol (protocol 2) causes rapid cooling and inactivation of enzymes, however, this procedure had only small advantages compared with protocol 3. Still, addition of ethanol allows in principle aliquoting of samples below 0 °C, as the melting of water/ethanol mixtures is below 0 °C. A downside to the addition of stabilizing agents such as ethanol is an additional step in the procedure and it may lead to distortions in metabolomics measurements such as reported for NMR (van der Sar et al. 2015). So despite the theoretical benefits of adding ethanol its positive effects seem minimal under these controlled sampling conditions and therefore not considered necessary for the class of metabolites studied. However, for more unstable metabolite classes the addition of ethanol could still be beneficial and needs further investigation.

The major strength of this study was that we were able to monitor ex vivo degradation of selected metabolites by adding them as isotopically-labeled markers to sampling tubes. Because of this addition we were also able to detect volume effects, which otherwise might have been mistaken for stability effects. A possible limitation of this labeling strategy is that some of the degradation of isotopically-labeled compounds occurred before CSF was added; additionally there might be some impurity of the isotopically-labeled standards that is within the limits of industrial quality. However, this would not affect the validity of our study, because preparation of the ILC-mix was performed in one batch, and for each subject we used one aliquot of the ILC-mix, which was thawed just before sampling. Furthermore, preparation of the sampling tubes with ILC-mix was highly standardized, and there were no differences between protocols in time between preparation of sampling tubes and CSF sampling. Nonetheless, if degradation of the ILC-mix had occurred prior to CSF sampling, this would have resulted in an overestimation of degradation; the observed degradation of ^13^C_2_-glutamine to ^13^C_2_-glutamic acid can therefore be considered an upper limit of degradation. The unique labeling strategy used in this study could benefit future studies addressing stability issues of other metabolites, because it allows detection of degradation during sampling and sample handling with a higher sensitivity and selectivity than just studying endogenous metabolites.

An important limitation of the study is that it does not fully reflect circumstances as they occur in clinical practice. In our study samples were processed relatively fast and were in the freezer within 2 h. In clinical practice samples often have to be transported, i.e. from the outpatient clinic to the laboratory for centrifugation or to special freezer locations. This study, therefore, does not provide information on effects of temperature, centrifugation or ethanol addition when sample processing takes longer than 2 h. Neither did we investigate effects of tube transfer or extra freeze–thaw cycles before metabolome analysis. Complications that can both occur in research and diagnostic settings and could be addressed in future studies.

## Conclusion

We have evaluated five different CSF sampling protocols, including an innovative sampling protocol which uses ethanol as preservative, on stability of endogenous amines. The effects of centrifugation, temperature and addition of ethanol were minimal for most amines, with the exception of PE, GABA and to a lesser extent l-glutamic acid. In addition, we showed that ex vivo degradation of l-glutamine to l-glutamic acid under controlled conditions is very limited. Taken together, we conclude that the consensus guideline for sampling CSF as was previously published (Del Campo et al. 2012) is adequate for most amines (at least for metabolites included in this study), provided that samples are cooled to 4 °C immediately after collection, centrifuged, and stored at − 80 °C within 2 h.

## Electronic supplementary material

Below is the link to the electronic supplementary material.

Supplementary material 1 (DOCX 135 KB)

## Abbreviations

CSF: Cerebrospinal fluid
LC–MS: Liquid chromatography–mass spectrometry
ILC: Isotopically-labeled compounds
ISTD: Internal standard
PE: *O*-phosphoethanolamine
GABA: Gamma-aminobutyric acid butyric acid
