# Supplementary material for: Strategies to assess and optimize stability of endogenous amines during cerebrospinal fluid sampling
Source: Metabolomics. 2018 Mar 5;14(4):44. doi: 10.1007/s11306-018-1333-0 (PMC5838118; doi:10.1007/s11306-018-1333-0)
Supplement: Supplementary file 1 — Supplementary material 1 (DOCX 135 KB) [file 11306_2018_1333_MOESM1_ESM.docx]

SUPPLEMENTARY INFORMATION

Strategies to assess and optimize stability of endogenous amines during cerebrospinal fluid sampling

Marek Noga^1^‡, Ronald Zielman^2^‡, Robin M. van Dongen^2^, Sabine Bos^1^, Gisela M. Terwindt^2^, Arn M.J.M. van den Maagdenberg^2,3^, Thomas Hankemeier^1^‡, Michel D. Ferrari^2^‡

^1^ Division of Analytical Biosciences, Leiden Academic Centre for Drug Research, Leiden, the Netherlands

^2^ Department of Neurology, Leiden University Medical Centre, Leiden, the Netherlands

^3^ Department of Human Genetics, Leiden University Medical Centre, Leiden, the Netherlands,

‡ These authors contributed equally to the manuscript

**Contents**

**Supplemental Methods and Figure S-1**: Volume correction page S-2

**Table S-1:** Endogenous amines without volume correction and without protocol 2 page S-4

**Table S-2:** Stability markers per protocol without volume correction and without protocol 2 page S-5

**Journal:** Metabolomics

Address for correspondence Thomas Hankemeier. Division of Analytical Biosciences, Leiden Academic Centre for Drug Research, Leiden University, Einsteinweg 55, 2333 CC Leiden, the Netherlands

Email: [hankemeier@lacdr.leidenuniv.nl](mailto:hankemeier@lacdr.leidenuniv.nl). Phone: +31 71 5274226

**Supplemental Methods: Volume correction**

In CSF samples from protocol 2 concentrations of almost every endogenous and isotopically-labeled metabolite were approximately 20% higher in comparison to samples from the other protocols (Fig. S-1a and c). The fact that both endogenous (from CSF) and isotopically-labeled metabolites (from ILC-mix) were increased indicated that there was less ethanol in samples from protocol 2 than anticipated. In order to test for differences in metabolite levels between all five protocols, without the influence of this systematic deviation, we standardized concentrations of all metabolites to the concentrations of alanine and isoleucine (Fig. S-1b and d)

**
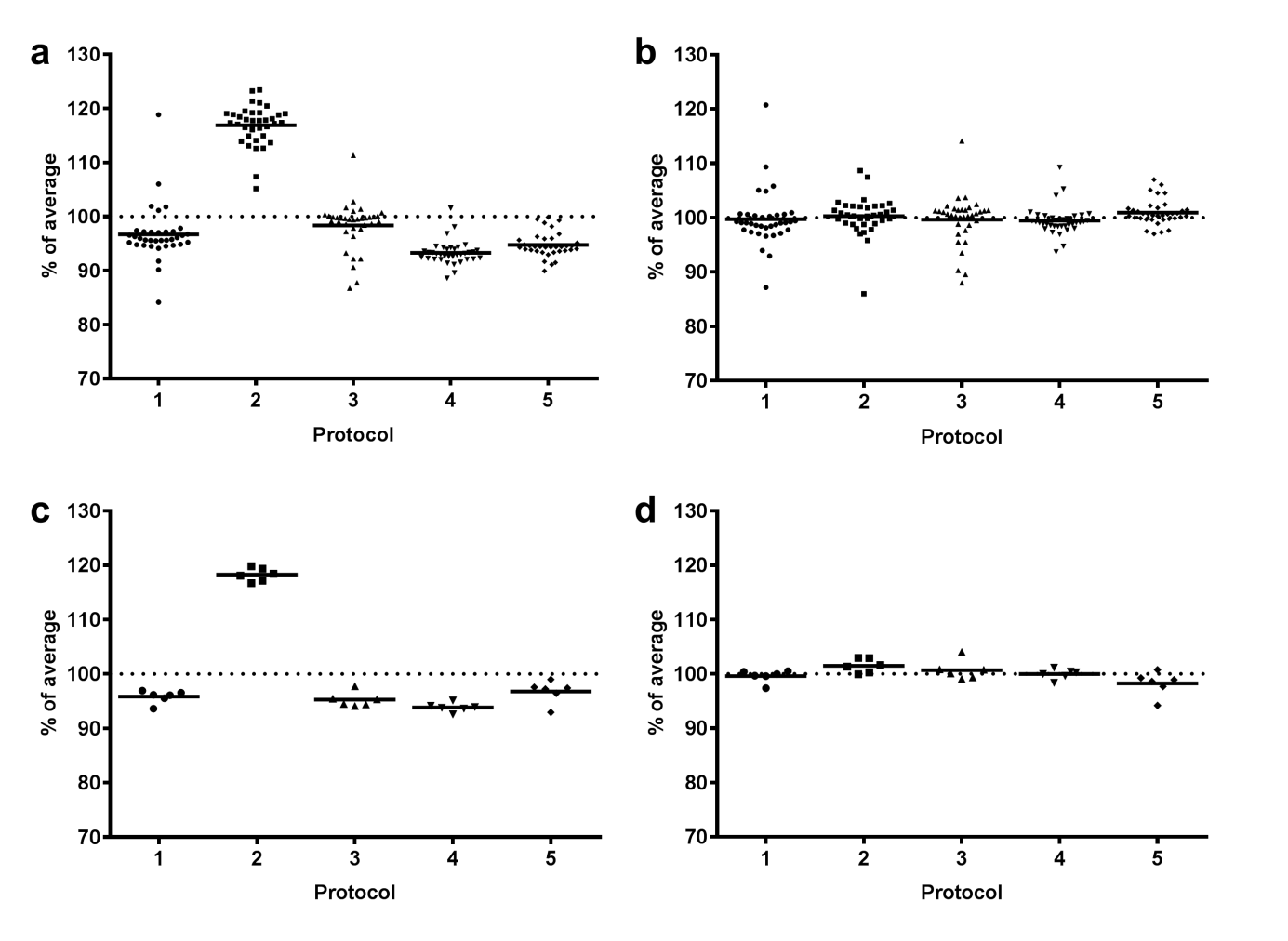
**

**Figure S-1. Differences between protocols before and after volume correction.** Every dot represents a compound. The average concentration of the compound is shown for each protocol (n=21 subjects) relative to the overall average (n=105; dotted lines at 100%). Before volume correction, all endogenous amines **(a)** and isotopically-labeled compounds **(c)** are higher in protocol 2. After volume correction, both endogenous amines **(b)** and isotopically-labeled compounds **(d)** are similar between protocols.

We standardized isotopically-labeled compounds according to protocol differences observed for ^13^C_2_-alanine and ^13^C^15^N-isoleucine because these compounds were considered to be stable.

**Step 1: General** Reference concentrations were calculated as follows:

^13^C_2_-alanine = average concentration of ^13^C_2_-alanine across n=21 reference samples (ILC-mix with 1 mL of water instead of CSF).

^13^C^15^N-isoleucine = average concentration of ^13^C^15^N-isoleucine across n=21 reference samples (ILC-mix with 1 mL of water instead of CSF).

*Reference concentrations of ^13^C_2_-alanine and ^13^C^15^N-isoleucine were 0.5996 and 0.3350, respectively.*

**Step 2: For each sample** a correction factor was calculated as follows:

^13^C_2_-alanine = sample concentration of ^13^C_2_-alanine / reference concentration of ^13^C_2_-alanine.

^13^C^15^N-isoleucine = sample concentration of ^13^C^15^N-isoleucine / reference concentration of ^13^C^15^N-isoleucine.

The mean of both numbers is the correction factor.

**Step 3: For each sample** the correction factor was applied as follows:

Corrected signal = observed concentration of compound X in sample Y / correction factor of sample Y.

**EXAMPLE**

Sample Y of protocol 2 has the following concentrations

^13^C_2_-alanine: 0.7089

^13^C^15^N-isoleucine: 0.4078

^13^C_2_-glutamine: 510.7

**Step 2**: The correction factor would then be (0.7090/0.5996 + 0.4078/0.3350)/2 = 1.120.

**Step 3:** Corrected concentration would be

^13^C_2_-alanine: 0.7089/1.120 = 0.632

^13^C^15^N-isoleucine: 0.4078/1.120 = 0.364

^13^C_2_-glutamine: 510.7 /1.120 = 456.0

For endogenous amines we applied the same calculation steps as above with L-alanine and L-isoleucine as reference. For each sample protocol 4 concentrations were chosen as reference concentrations.

**Table S-1.** **Endogenous amines without volume correction and without protocol** **2**

| **Metabolites** | **Relative concentration** | | | | **p-value** |
| --- | --- | --- | --- | --- | --- |
|  | **Protocol 1** | **Protocol 3** | **Protocol 4** | **Protocol 5** |  |
| gamma-aminobutyric acid | 0.0071 ± 0.0026 | 0.0051 ± 0.002 | 0.0053 ± 0.0021 | 0.0056 ± 0.0023 | **<0.001** |
| o-phosphoethanolamine | 0.078 ± 0.02 | 0.066 ± 0.019 | 0.076 ± 0.019 | 0.078 ± 0.019 | **0.010** |
| L-glutamic acid | 0.022 ± 0.009 | 0.018 ± 0.009 | 0.019 ± 0.006 | 0.02 ± 0.01 | **0.017** |
| L-arginine | 1.896 ± 0.458 | 2.004 ± 0.459 | 1.828 ± 0.447 | 1.918 ± 0.455 | **0.048** |
| putrescine | 0.0082 ± 0.0025 | 0.0085 ± 0.0031 | 0.0077 ± 0.003 | 0.0082 ± 0.003 | 0.092 |
| ADMA | 0.0005 ± 0.0002 | 0.0007 ± 0.0003 | 0.0006 ± 0.0003 | 0.0006 ± 0.0003 | 0.097 |
| L-phenylalanine | 0.023 ± 0.004 | 0.023 ± 0.005 | 0.022 ± 0.005 | 0.022 ± 0.005 | 0.144 |
| SDMA | 0.004 ± 0.001 | 0.004 ± 0.001 | 0.004 ± 0.001 | 0.004 ± 0.001 | 0.159 |
| L-tryptophan | 0.31 ± 0.064 | 0.329 ± 0.077 | 0.302 ± 0.071 | 0.313 ± 0.07 | 0.159 |
| L-methionine sulfoxide | 0.016 ± 0.005 | 0.014 ± 0.005 | 0.015 ± 0.005 | 0.014 ± 0.005 | 0.178 |
| L-methionine | 0.537 ± 0.123 | 0.565 ± 0.161 | 0.517 ± 0.124 | 0.532 ± 0.119 | 0.189 |
| L-tyrosine | 0.725 ± 0.154 | 0.758 ± 0.177 | 0.708 ± 0.166 | 0.734 ± 0.181 | 0.213 |
| ethanolamine | 3.595 ± 0.562 | 3.786 ± 0.726 | 3.611 ± 0.688 | 3.745 ± 0.63 | 0.279 |
| taurine | 0.302 ± 0.085 | 0.319 ± 0.092 | 0.296 ± 0.085 | 0.306 ± 0.088 | 0.289 |
| L-glutamine | 11.877 ± 2.061 | 12.552 ± 2.582 | 11.754 ± 2.256 | 12.094 ± 2.693 | 0.326 |
| L-valine | 0.037 ± 0.011 | 0.038 ± 0.013 | 0.035 ± 0.011 | 0.037 ± 0.012 | 0.328 |
| L-alanine | 0.068 ± 0.016 | 0.07 ± 0.019 | 0.066 ± 0.018 | 0.067 ± 0.016 | 0.375 |
| L-kynurenine | 0.0023 ± 0.0012 | 0.0023 ± 0.0009 | 0.0023 ± 0.0011 | 0.0025 ± 0.0009 | 0.438 |
| L-leucine | 0.018 ± 0.004 | 0.018 ± 0.005 | 0.017 ± 0.005 | 0.017 ± 0.005 | 0.444 |
| L-asparagine | 0.322 ± 0.073 | 0.331 ± 0.081 | 0.314 ± 0.088 | 0.319 ± 0.073 | 0.448 |
| L-4-hydroxyproline | 0.023 ± 0.013 | 0.024 ± 0.015 | 0.022 ± 0.013 | 0.023 ± 0.015 | 0.454 |
| citrulline | 0.04 ± 0.014 | 0.041 ± 0.017 | 0.038 ± 0.015 | 0.04 ± 0.017 | 0.462 |
| L-histidine | 0.09 ± 0.016 | 0.094 ± 0.019 | 0.089 ± 0.021 | 0.092 ± 0.018 | 0.477 |
| L-aspartic acid | 0.055 ± 0.051 | 0.054 ± 0.057 | 0.052 ± 0.043 | 0.051 ± 0.056 | 0.508 |
| L-alpha-aminobutyric acid | 0.072 ± 0.021 | 0.077 ± 0.034 | 0.07 ± 0.022 | 0.073 ± 0.025 | 0.532 |
| L.Lysine | 3.221 ± 0.787 | 3.337 ± 0.793 | 3.137 ± 0.754 | 3.244 ± 0.793 | 0.541 |
| N6N6N6-trimethyl-L-lysine | 0.0056 ± 0.0008 | 0.0059 ± 0.0013 | 0.0057 ± 0.0013 | 0.0059 ± 0.0011 | 0.549 |
| L-threonine | 1.359 ± 0.359 | 1.414 ± 0.45 | 1.324 ± 0.399 | 1.361 ± 0.357 | 0.614 |
| glycylglycine | 0.021 ± 0.008 | 0.02 ± 0.009 | 0.022 ± 0.015 | 0.022 ± 0.008 | 0.722 |
| L-serine | 2.574 ± 0.665 | 2.66 ± 0.7 | 2.534 ± 0.658 | 2.607 ± 0.67 | 0.740 |
| L-proline | 0.025 ± 0.018 | 0.026 ± 0.02 | 0.024 ± 0.015 | 0.024 ± 0.017 | 0.760 |
| ornithine | 0.2 ± 0.071 | 0.202 ± 0.08 | 0.192 ± 0.069 | 0.196 ± 0.072 | 0.818 |
| L-2-aminoadipic acid | 0.0011 ± 0.0004 | 0.0011 ± 0.0004 | 0.0011 ± 0.0004 | 0.001 ± 0.0003 | 0.830 |
| L-isoleucine | 0.013 ± 0.004 | 0.013 ± 0.004 | 0.013 ± 0.004 | 0.013 ± 0.004 | 0.833 |
| sarcosine | 0.001 ± 0.0003 | 0.001 ± 0.0005 | 0.0011 ± 0.0005 | 0.0011 ± 0.0005 | 0.850 |
| L-homoserine | 0.0061 ± 0.0014 | 0.006 ± 0.0016 | 0.0058 ± 0.0018 | 0.0059 ± 0.0012 | 0.853 |

Relative concentrations reported as response ratios to their respective internal standards. P-values from one-way repeated measures ANOVA. Metabolites sorted based on p-value (small to large). P-values < 0.05 are depicted in bold. SDMA = symmetric dimethylarginine, ADMA = asymmetric dimethylarginine.

**Table S-2.** **Stability markers per protocol without volume correction and without protocol 2**

| **Metabolites** | **Absolute concentration (µmol/L)** | | | | **p-value** |
| --- | --- | --- | --- | --- | --- |
|  | **Protocol 1** | **Protocol 3** | **Protocol 4** | **Protocol 5** |  |
| ***Stable*** |  |  |  |  |  |
| 2,3-^13^C_2_-alanine | 25.67 ± 4.21 | 25.23 ± 4.6 | 25.04 ± 3.84 | 26.28 ± 3.97 | 0.480 |
| ^13^C^15^N-isoleucine | 5.53 ± 0.97 | 5.46 ± 1.07 | 5.40 ± 0.86 | 5.70 ± 0.89 | 0.476 |
|  |  |  |  |  |  |
| ***Unstable*** |  |  |  |  |  |
| 1,3-^13^C_2_-glutamine | 404 ± 75 | 398 ± 80 | 394 ± 67 | 412 ± 70 | 0.564 |
| ^13^C_2_-glutamic acid | 0.64 ± 0.11 | 0.63 ± 0.14 | 0.65 ± 0.13 | 0.68 ± 0.11 | 0.233 |
| U^13^C-U^15^N-UD-glutamic acid | 10.19 ± 1.84 | 10.02 ± 2.44 | 10.04 ± 1.65 | 10.59 ± 1.75 | 0.397 |
| 1,2,3,4,5,6-^13^C_6_-dopamine | 0.09 ± 0.02 | 0.1 ± 0.02 | 0.09 ± 0.01 | 0.09 ± 0.01 | 0.799 |
|  |  |  |  |  |  |
| ***Degradation* ^a^** | **Conversion from glutamine to glutamate (%)** | | | |  |
| 1,3-^13^C_2_-glutamine to  ^13^C_2_-glutamic acid (%) | 0.158 ± 0.012 | 0.160 ± 0.027 | 0.165 ± 0.015 | 0.166 ± 0.017 | 0.191 |

P-values from one-way repeated measures ANOVA. ^a^ Volume correction was not necessary since both glutamine and glutamate were measured in the same volume.
